# Photocatalytic Material–Microbe Hybrids: Applications in Environmental Remediations

**DOI:** 10.3389/fbioe.2021.815181

**Published:** 2022-01-31

**Authors:** Yadong Yu, Shanshan Wang, Jinrui Teng, Anze Zupanic, Shuxian Guo, Xiaobin Tang, Heng Liang

**Affiliations:** ^1^ College of Biotechnology and Pharmaceutical Engineering, Nanjing Tech University, Nanjing, China; ^2^ 2011 College, Nanjing Tech University, Nanjing, China; ^3^ Department of Biotechnology and Systems Biology, National Institute of Biology, Ljubljana, Slovenia; ^4^ Henan Key Laboratory of Industrial Microbial Resources and Fermentation Technology, Nanyang Institute of Technology, Nanyang, China; ^5^ State Key Laboratory of Urban Water Resource and Environment, Harbin Institute of Technology, Harbin, China

**Keywords:** photocatalytic material, microbe, hybrid, environmental remediation, solar power

## Abstract

Environmental pollution has become one of the most urgent global issues that we have to face now. Searching new technologies to solve environmental issues is of great significance. By intimately coupling photocatalytic materials with microbes, the emerging photocatalytic material–microbe hybrid (PMH) system takes advantages of the high-efficiency, broad-spectrum light capture capability of the photocatalytic material and the selectivity of microbial enzymatic catalysis to efficiently convert solar energy into chemical energy. The PMH system is originally applied for the solar-to-chemical production. Interestingly, recent studies demonstrate that this system also has great potential in treating environmental contaminations. The photogenerated electrons produced by the PMH system can reductively decompose organic pollutants with oxidative nature (e.g., refractory azo dyes) under anaerobic circumstances. Moreover, based on the redox reactions occurring on the surface of photocatalysts and the enzymatic reactions in microbes, the PMH system can convert the valences of multiple heavy metal ions into less toxic or even nontoxic status simultaneously. In this review, we introduce the recent advances of using the PMH system in treating environmental pollutions and compare this system with another similar system, the traditional intimately coupled photocatalysis and biodegradation (ICPB) system. Finally, the current challenges and future directions in this field are discussed as well.

## Introduction

Environmental pollution is one of the world’s most severe problems that we have to face nowadays. This has caused great concern globally, and it is urgent to develop more efficient methods to handle environmental issues. Photocatalytic oxidation and microbe bioremediation are considered as two promising methods and have been extensively explored (Lin et al., 2012; Karim et al., 2021). Photocatalytic oxidation-based approaches mainly utilize the photogenerated holes and electrons on the surfaces of photocatalytic materials to generate reactive oxygen species (ROS), which will then oxidatively decompose pollutants (Byrne et al., 2018). Photocatalytic oxidation provides the possibility of utilizing sustainable and green solar energy to remove pollutants. As for microbe-based bioremediation, pollutants are removed by the versatile catabolic processes in microbes, and thereby, this method is cost-effective and eco-friendly (Juan-Luis et al., 2011).

Recently, photocatalytic materials and microbes are intimately combined together to form the so-called photocatalytic material–microbe hybrid (PMH) system (Du et al., 2020). In 2016, the PMH system is firstly built by Professor Peidong Yang’ group in Berkeley, University of California, to realize solar-to-chemical production (Sakimoto et al., 2016). The PMH system combines the advantages of abiotic catalysis and biotransformation for the production of value-added chemicals and fuels. Photocatalytic materials sustainably provide reducing power from light to microbe, which then uses these reducing equivalents to synthesize products. Currently, PMH systems are mainly applied for H_2_ evolution (Jiang et al., 2018; Wei et al., 2018), CO_2_ conversion (Ye et al., 2019), N_2_ fixation (Wang B. et al., 2019; Chen et al., 2019), and chemical manufacturing (Guo et al., 2018; Zhang et al., 2020).

Interestingly, this PMH system has also shown great potential in treating environmental pollutants in some very recent reports (Wang X. et al., 2019). Yet, to the best of our knowledge, there is no review summarizing these advances. Therefore, we summarize the current advances of using the PMH system in environmental remediation and compare it with another similar system, the intimately coupled photocatalysis and biodegradation (ICPB). Current research gaps and future perspectives are also given in the end. We believe this review will be helpful for the future development of this direction.

## Application of PMHs in Environmental Remediation

**FIGURE 1:**
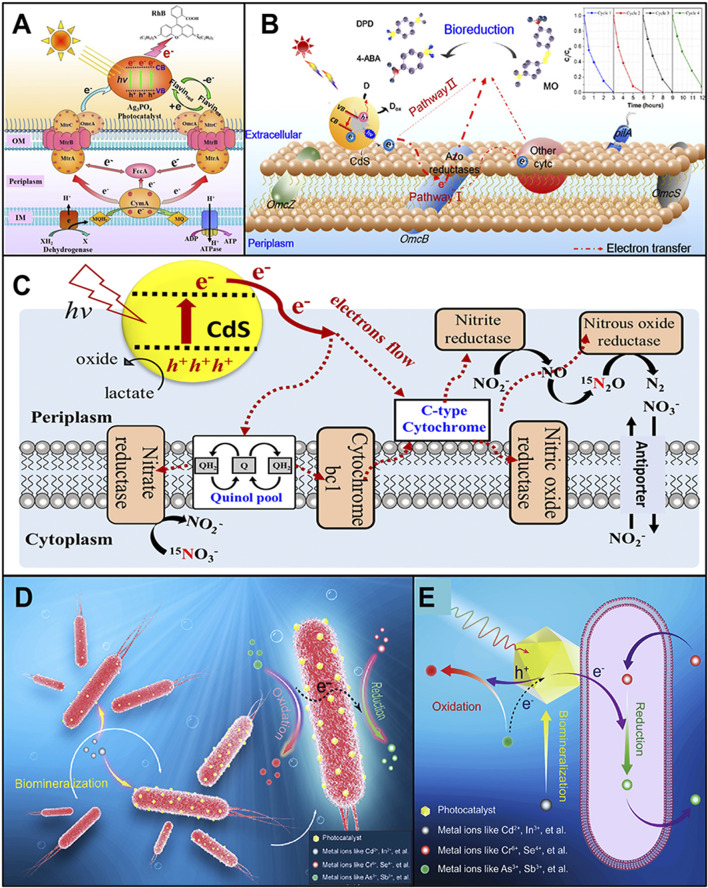
**(A)** Schematic illustration of the light-driven degradation pathway of rhodamine B using the Ag_3_PO_4_–*S. oneidensis* MR-1 hybrid system (reprinted with permission from Xiao et al., 2019). **(B)** Schematic illustration of the degradation pathway of MO using the CdS–*G. sulfurreducens* hybrid system (reprinted with permission from Huang et al., 2019). **(C)** Schematic illustration of the denitrification mechanism in the CdS–*T. denitrificans* hybrid system driven by light (reprinted with permission from Chen et al., 2019). **(D**, **E)** Mechanism of fabricating the CdS nanomaterial–*B. thuringiensis* HM-311 hybrid based on the microbial mineralization ability **(D)** and the electron transfer between different heavy metal ions in the hybrid system **(E)** (reprinted with permission from Zuo et al., 2021a).

Most organic contaminants can be gradually biodegraded and completely mineralized through the aerobic respiration of microbes. Yet, the conventional aerobic treatment increases the energy consumption because of the high aeration rate during the operation process (Mitsugu and Yasumoto, 2003). In addition, some pollutants with oxidative nature (such as refractory azo dyes and halogenated organic and nitroaromatic compounds) tend to be degraded reductively rather than oxidatively (Liu P.-C. et al., 2020). Therefore, it is meaningful to develop new technologies which can provide adequate reducing power to fulfill the reduction degradation of these pollutants under anaerobic conditions. In this context, Xiao et al. (2019) coupled an electrochemically active bacterium, *Shewanella oneidensis* MR-1, with the Ag_3_PO_4_ photocatalyst to build a PMH system, which can remove almost 100% of 15 mg/L rhodamine B after 7 days under visible light irradiation. Blocking the Mtr respiratory pathway (a transmembrane electron transport chain) or dosing additional riboflavin (an electron shuttle) could interfere with the extracellular electron transfer of *S. oneidensis* MR-1 and decrease the degradation rate of rhodamine B. The authors believed that the photogenerated holes on the Ag_3_PO_4_ photocatalyst were scavenged by the electrons released from *S. oneidensis* MR-1, and thereby, the photogenerated electrons on the Ag_3_PO_4_ photocatalyst were efficiently transferred to rhodamine B and achieve the photo-reductive degradation (Figure 1A). Xiao et al. also used this PMH system to reductively decompose *p*-chlorophenol under anaerobic circumstances. The results of density functional theory calculations further proved that the electrochemically active bacterium *S. oneidensis* MR-1 could act as a biological hole scavenger (Liu P.-C. et al., 2020). In another of their work, they used CdS nanocrystals instead of Ag_3_PO_4_ to combine with *S. oneidensis* MR-1 and form a PMH system for the treatment of trypan blue (Xiao et al., 2020). Again, the biogenic electrons produced by the anaerobic respiration of *S. oneidensis* MR-1 eliminated the photogenerated holes on CdS nanocrystals and maintained the ongoing photoexcitation to produce electrons, cleaving the azo bonds in trypan blue.

Similarly, to degrade another azo dye, methyl orange (MO), Huang et al. (2019) built a PMH system by precipitating CdS nanoparticles on the surface of *Geobacter sulfurreducens* (an electrochemically active bacterium). This PMH system can completely degrade 40 mg/L MO after 3 h under light irradiation. Different from the mechanism proposed by Liu et al. (2020b) and Xiao et al. (2019, 2020), Huang et al. believed that there were two degradation pathways working simultaneously in their PMH system (Figure 1B): (1) the photogenerated electrons were stored on the outer-membrane cytochrome C of *G. sulfurreducens*, and these electrons were then transferred to the terminal reductase for mineralizing MO; and (2) the photogenerated electrons could be directly transferred to the MO and degrade it. Huang et al. (2019) hypothesized that the photogenerated holes were scavenged by the intermediates produced during the decolorization process of MO as they found that dosing additional sacrificial reagents in the PMH system did not affect the MO degradation rate. In the report contributed by Chen et al. (2019), a PMH system containing CdS nanomaterials and *Thiobacillus denitrificans* was used to supply electrons for the denitrification process. After 68 h of visible light irradiation, this PMH system converted more than 72.1% of the NO_3_^−^-N into N_2_O-N. Lactate was chosen as the sacrificial reagent to scavenge the photogenerated holes. The authors proposed that the excited photoelectrons from CdS nanomaterials were transferred into microbes through the electron acceptors in cell membranes like C-type cytochrome and then to those enzymes involved in the denitrification pathway (Figure 1C).

Apart from persistent organic pollutants, the PMH system can also be used to treat heavy metal contamination. Our group deposited CdS nanomaterials on the surface of *Bacillus thuringiensis* HM-311 (a multiple-heavy-metal-tolerant strain isolated from heavy-metal-polluted soil (Berrazeg et al., 2019)) to form the PMH system (Zuo et al., 2021b). Then this PMH system was operated in the simulated Cr^6+^ (100 mg/L) and As^3+^ (100 mg/L) co-contaminated water under visible light. After 24 h of operation, 99.25% of Cr^6+^ was reduced. Meanwhile, As^3+^ was simultaneously oxidized into As^5+^ by this PMH system, and up to 62.74 mg/L of As^5+^ was detected. We believed that with the help of solar energy and microbial enzymatic reactions, electrons can be transferred from heavy metal ions that need to be oxidized to detoxify them and donated to heavy metal ions that need to be reduced to become less toxic (Figures 1D,E). This PMH system might realize the electron transfer between different heavy metal ions, achieving the repair of multiple metal ions in wastewater simultaneously.

## Compare PMH With ICPB

In fact, it is not the first time that photocatalysts are combined with microbes intimately for environmental remediation. The group led by Professor Rittmann at Arizona State University first built a system which intimately coupled photocatalytic oxidation with microbial degradation (ICPB) for wastewater treatment in 2008 (Marsolek et al., 2010). Herein, we compare the ICPB system with the PMH system. This will help researchers understand the differences and connections between these two systems and apply them more properly for wastewater treatment.

### Mechanisms and Target Pollutants

A typical ICPB system mainly consists of porous carriers, photocatalysts, biofilms, and an illuminated reactor. Photocatalysts are loaded on the surface of the carriers, and the biofilms are cultivated in the carriers. The porous carrier can adsorb refractory pollutants from the solution and enhance its mass transfer to the surface of the photocatalysts. In the process of photocatalytic degradation, a photocatalyst absorbs photon energy and generates electron–hole pairs, which will cause ROS such as •OH and •O_2_^−^. These ROS will attack and destroy the refractory organic contaminants and convert them into biodegradable products, which are completely degraded by the biofilms afterwards (Liu J. et al., 2020; Wu et al., 2020). ICPB systems are suitable to mineralize most of the refractory organic pollutants (e.g., antibiotics, dyes, and polycyclic aromatic hydrocarbons). A typical PMH system is mainly composed of microbial cells and photocatalytic materials deposited directly on their surfaces. For environmental remediation, electrochemically active bacteria are often used in PMH systems to provide the biogenic electrons to eliminate the photogenerated holes on the photocatalysts. In this case, the photogenerated electrons are able to reductively degrade those organic pollutants with oxidative natures (such as nitroaromatic compounds and azo dyes). In the PMH system for treating heavy metals, electrons generated by photocatalysts can be transferred into microbial cells to promote intracellular enzymatic reductions, reducing heavy metal ions such as Cr^6+^ and Se^4+^. Heavy metal ions like As^3+^ and Sb^3+^ can be oxidized by photogenerated holes as sacrificial electron donors.

### Light Source

In the PMH system, additional carriers are not required as photocatalysts are directly coated on the surface of bacteria. Considering that most microbes cannot survive under ultraviolet (UV) light irradiation, the PMH system is often driven by visible light. For the ICPB system, the excitation light can be UV light or visible light since the biofilms are protected by the carriers. Yet, we have to point out that although UV-light-excited photocatalysts display good efficiencies in ICPB, their practical applications are still not widespread because UV light accounts for only 4% of sunlight (Zhang et al., 2016). Plus, UV light exhibits a strong killing ability on microorganisms. Under UV light irradiation, biofilms are detached from the carriers and the soluble microbial products are generated, which increase the turbidity of the reaction mixture. This could in turn hinder the penetration of UV light and thereby reduce the photocatalytic degradation efficiency (Zuo et al., 2021a). Therefore, most ICPB systems are driven by visible light now.

### Microbes

In the ICPB system, microbes are the implementers of biodegradation. It could be single strain with the ability to degrade the products from the photooxidation stage. Yet, more ICPB systems choose the microbial consortium as the implementers of biodegradation because it is hard to completely degrade organic pollutants by one microbial strain. Microbial consortium contains different types of microorganisms, can corporate with each other, and show a synergy effect when they degrade organic pollutants. Thus, the microbial consortium can degrade different types of organic pollutants and their toxic intermediate metabolites, achieving a complete-degradation effect. In the PMH systems designed for removing organic pollutants, electrochemically active bacteria are usually chosen. These bacteria can provide bio-electrons *via* their anaerobic respiration to scavenge the photogenerated holes on the photocatalysts and maintain the ongoing photoreductive degradation of those oxidative organic pollutants. These bacteria are not capable of decomposing the intermediates produced from the photoreductive degradation process.

### Electron Donors

For ICPB systems, microbes are key degraders which completely mineralize the photocatalysis products. However, this biodegradation process could be compromised if the photocatalysis products remain refractory or inhibitory. Providing readily biodegradable co-substrates such as acetate which could act as extra electron donors and energy sources will improve the microbial metabolic activity, counteract toxicity effects, and accelerate oxygenation reactions, resulting in a great increase of the removal rate (Bai et al., 2015; Xiong et al., 2018). In PMH systems, photogenerated holes need to be scavenged, which will ensure the system will provide reductive electrons for microbial synthesis or the photoreductive degradation of target pollutants. For those PMH systems used for environmental purposes, the hole scavengers are the electrochemically active bacteria, which need carbon sources and electron donors such as lactate and sodium acetate trihydrate. In our previous work, some heavy metal ions (e.g., As^3+^ and Sb^3+^) can be used as sacrificial electron donors to maintain the continuous operation of the PMH system (Zuo et al., 2021b).

### Operation Conditions

**FIGURE 2:**
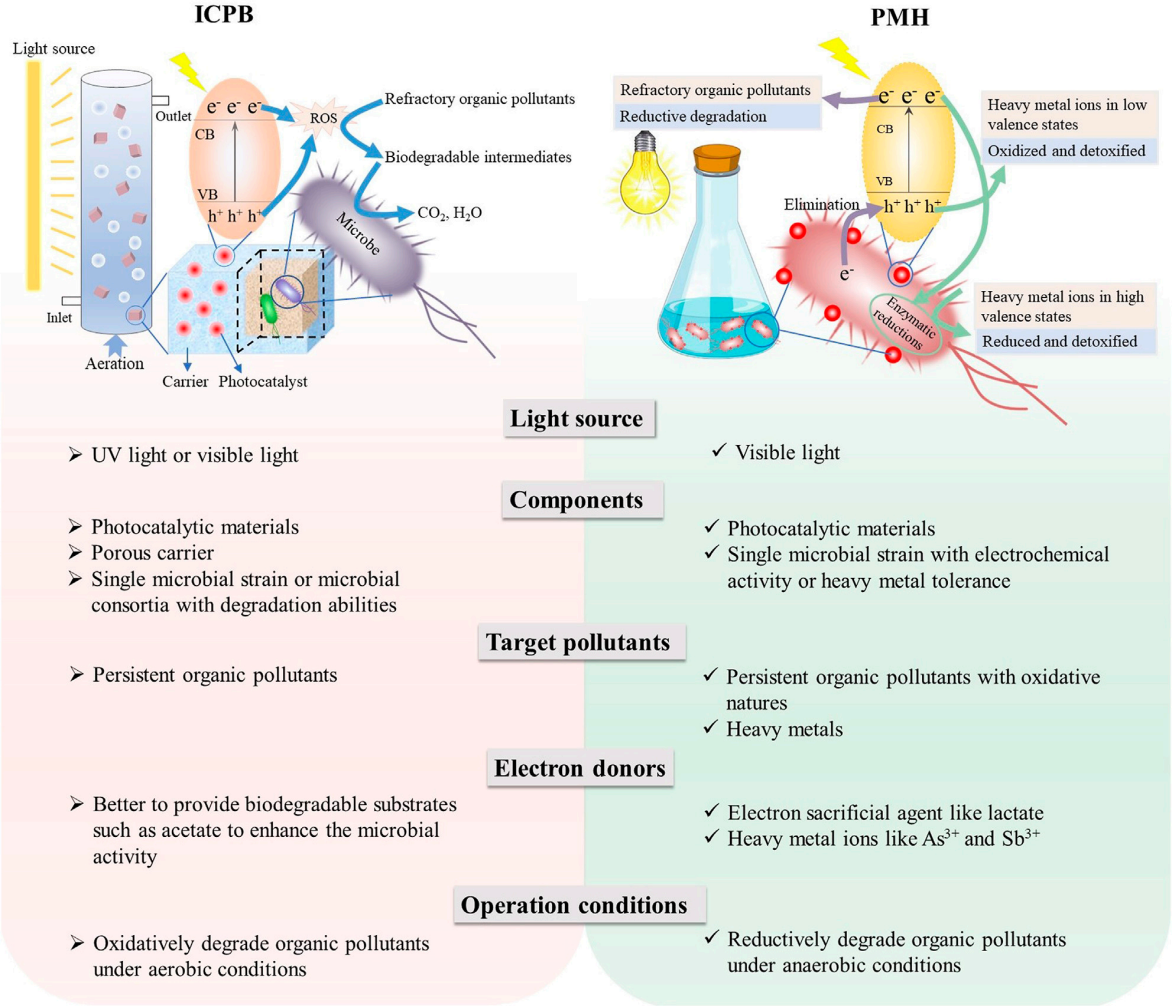
Comparisons between the ICPB system and PMH system.

As shown in Figure 2, the ICPB system is agitated and operated in an illuminated reactor coupled with an aeration pump, which supplies enough oxygen for the photocatalysis oxidation and microbial respiration. In contrast, PMH systems can work under anaerobic conditions to achieve photoreductive degradation of those oxidative organic pollutants. Since it does not need to aerate, the PMH system decreases the energy consumption during the operation process.

## Conclusions and Perspective

Herein, we summarize the recent advances of using the PMH systems for environmental remediations. Driven by sustainable, green sunlight, these emerging PMH systems may provide new and promising alternatives for anaerobically photoreductive decomposing organic pollutants with oxidative natures and repairing multiple heavy metal ions simultaneously in wastewater. While some exciting results have already been obtained, the PMH system is still in its infancy, and some gaps that need more effort to address remain:

1) The photocatalytic materials used in PMH systems now often contain heavy metals or noble metals (e.g., CdS and Ag_3_PO_4_); more investigations are needed to address their biocompatibility, cost, and environmental impacts before implementing real environmental applications. Interestingly, some microbes can utilize natural extracellular semiconducting minerals (e.g., iron and manganese) to synthesize photosensitizers, which can decorate microbial membranes, facilitate the transmembrane electron transfer, and achieve light harvesting (Lu et al., 2019; Yu and Leadbetter, 2020). Thus, it might be a promising direction to synthesize earth-abundant semiconductor minerals as compatible photosensitizers for PMH systems based on the microbes’ biomineralization capabilities.
2) Microorganisms are the other key component in the PMH system. It is critical to improve the microbes’ capability to yield and transfer electrons, which requires us to discover and redesign the corresponding molecular pathways rationally in the future. The incorporation of more biology scientists especially with omics and synthetic biology backgrounds will be great.
3) Electron donors, either for the microbial growth or for the scavenging of the photogenerated holes on the photocatalysts directly, are crucial for the continuous operation of PMH systems. The costs of those typical hole scavengers such as cysteine, ascorbic acid, and HEPES are too high for large-scale application. Finding electron donors that can be regenerated and have low cost, as well as being biocompatible, will be of great benefit to ensure economic viability and applicability of PMH systems in environmental applications.
